# Prognostic implication of *TERT* promoter mutation and circulating tumor cells in muscle-invasive bladder cancer

**DOI:** 10.1007/s00345-022-04061-9

**Published:** 2022-06-17

**Authors:** Raquel Carrasco, Mercedes Ingelmo-Torres, Ascensión Gómez, Fiorella L. Roldán, Natalia Segura, María José Ribal, Antonio Alcaraz, Laura Izquierdo, Lourdes Mengual

**Affiliations:** 1 Present Address: Laboratori i Servei d Urologia, Hospital Clínic de Barcelona, Barcelona, Spain; 2 Institut d’Investigacions Biomèdiques August Pi i Sunyer, IDIBAPS, Barcelona, Spain; 3 Departament de Cirurgia i Especialitats Medicoquirúrgiques, Facultat de Medicina i Ciències de la Salut, Universitat de Barcelona (UB), Barcelona, Spain; 4 Departament de Biomedicina, Facultat de Medicina i Ciències de la Salut, Universitat de Barcelona (UB), Barcelona, Spain

**Keywords:** Biomarker, Bladder cancer, Circulating tumor cells, Liquid biopsy, Prognosis, *TERT* promoter mutation

## Abstract

**Purpose:**

Current clinical prognostic factors are not accurate enough to identify and monitor those muscle-invasive bladder cancer (MIBC) patients at high risk of progression after radical cystectomy (RC). Here, we determined genetic alterations in the tumor and circulating tumor cell (CTC) enumeration to find biomarkers useful for the management of MIBC after RC.

**Methods:**

Thirty-nine MIBC patients undergoing RC were included. Tumoral tissue DNA was analyzed by next generation sequencing. CTCs were isolated from blood collected before RC and one, four and 12 months later.

**Results:**

Sixteen (41%) patients progressed in a median time of 8.5 months and 11 (69%) of these patients harbored the *TERT* c.-124C > T mutation. All progressive patients harboring the *TERT* c.-124C > T mutation presented a significant increase in CTC number 12 months after RC compared to those without the mutation. Additionally, CTC number at 12 months was identified as an independent prognostic biomarker for tumor progression and cancer specific survival (CSS). Ten (63%) progressive patients showed an increment of CTC number with a median anticipation period of four months compared with imaging techniques.

**Conclusions:**

The *TERT* c.-124C > T mutation could be considered a biomarker of aggressivity. CTC enumeration is a useful tool for identifying MIBC patients at high risk of progression and CSS after RC and for detecting tumor progression earlier than imaging techniques.

**Supplementary Information:**

The online version contains supplementary material available at 10.1007/s00345-022-04061-9.

## Introduction

Approximately 25% of bladder cancers (BC) are diagnosed as muscle-invasive bladder cancer (MIBC). Radical cystectomy (RC) with lymphadenectomy is the standard treatment for localized MIBC. However, despite undergoing radical surgery, around 50% of MIBC patients will develop local relapse or distant metastasis within two years of RC [1]. The current classification of MIBC is unable to individually predict which patients will relapse and develop metastases and when these events will occur during patient follow-up.

MIBC is an aggressive tumor with a high genetic instability and mutation rate in genes involved in transcription, chromatin regulation and the cell cycle [2]. The most common event described in BC to date is point mutations of the telomerase reverse transcriptase (*TERT*) promoter, present in approximately 80% of tumors, regardless of grade and stage [3]. However, due to the genetic heterogeneity of BC, there is no predictive or prognostic molecular information for its clinical application.

On the other hand, liquid biopsy has been recently used instead of tumor tissue to explore diagnostic, prognostic and predictive biomarkers in several tumors, including bladder cancer [4–7]. It is believed that circulating tumor cells (CTCs) present in liquid biopsy represent metastatic precursors that have an important role in disease invasion and progression [5]. In fact, several studies demonstrated that CTC number in peripheral blood correlates with poor outcome [5–8].

Both tumor tissue and liquid biopsy represent a source of prognostic biomarkers that could have an impact on MIBC patient management. Here, we evaluated DNA mutations in tumor samples from patients who underwent RC and enumerated the CTCs in their blood samples at different time points during their follow-up after RC to identify biomarkers predicting a high risk of recurrence and progression.

## Materials and methods

### Patients and samples

A total of 39 consecutive MIBC patients [median age (range) 70 years (51–85); 31 males, 8 females] who underwent RC and extended lymphadenectomy between 2018 and 2019 at our center were prospectively included. The clinicopathological features of the patients enrolled are summarized in Supplementary Table S1. Follow-up data was available for all patients (Supplementary Methods).

In our cohort, 8 of the 39 MIBC patients received neoadjuvant chemotherapy (NAC), and the remaining 31 MIBC patients did not for the following reasons: 11 patients were unfit for the NAC criteria, and 20 patients could not delay cystectomy.

Tissue samples were obtained from cystectomy specimens (*N* = 31). In case of pT0 in the cystectomy specimen, samples were obtained from transurethral resection of bladder tumor (TURBT) that showed muscle-invasive disease (*N* = 7). A tissue sample was not available for one patient (Patient 10) in the tumor biobank.

One 10 mL EDTA tube of peripheral blood was collected before RC and at one, four and 12 months after surgery. Blood samples were stored at room temperature until processed within the following 24 h.

### Next generation sequencing (NGS) of tumoral tissue

A total of 38 tissue samples were analyzed by NGS using the Ion Torrent Oncomine Comprehensive Assay v3 (Thermo Fisher Scientific, Massachusetts, USA) (Supplementary Methods and Supplementary Table S2). NGS data was analyzed with Ion Reporter Software v5.12 (Thermo Fisher Scientific) using a Oncomine Extended (5.18) filter to identify pathological mutations. Statistical analysis is detailed in Supplementary Methods.

### CTC isolation and enumeration

CTCs from blood samples were isolated via the IsoFlux system (Fluxion, Biosciences) and stored at 4ºC until enumeration within the following two weeks (Supplementary Methods). CTCs were fixed and immunofluorescence stained using the CTC Enumeration Kit (Fluxion, Biosciences), following manufacturer’s instructions. CTC enumeration was performed manually using fluorescence microscopy (Supplementary Methods and Supplementary Figure S1). Molecular progression during follow-up was defined as the increase of at least 10 CTCs per 7.5 mL blood between two follow-up points. Statistical analysis is detailed in Supplementary Methods.

## Results

### Clinicopathological features of the cohort

Four of the 39 patients included in this study presented positive lymph nodes (LN +) at MIBC diagnosis (Supplementary Table S1). During a median follow-up of 25.5 months, 16 (41%) patients progressed (two with LN +). The median time to progression was 8.5 months (range 1–16 months). Overall, five of the 16 patients who progressed received neoadjuvant or adjuvant chemotherapy (one < pT2 and four pT3-4). The remaining seven pT3-4 patients had comorbidities or did not consent to neoadjuvant or adjuvant chemotherapy. Of the progressive patients, 81.3% (13/16) received treatment upon tumor progression. During follow-up, 15 (38.5%) patients died; 12 (80%) due to MIBC. Overall, 6.7% of the patients who died had LN + . Eight of the 12 (67%) patients who died had pT3 and pT4 tumors at MIBC diagnosis. The median time of cancer specific survival (CSS) was 15 months (range 4–31 months).

Four non-progressive patients developed another primary tumor during follow-up. These tumors were sarcoma, upper tract urothelial carcinoma, lung and ovarian cancers.

### Mutations in tumor samples

A median of five (range 1–34) mutations were identified in bladder tumor samples (Supplementary Table S3). *ATM, TP53* and *TERT* were the most frequently mutated genes in our cohort. Notably, the c.-124C > T hotspot mutation in the *TERT* promoter was found in 66% (25/38) of cases. Another *TERT* promoter mutation (c.-146C > T), the *ATM* c.1236-2A > T mutation and the *TP53* c.853G > A mutation were found in 16%, 42% and 13% of samples, respectively. Interestingly, 92% of MIBC samples analyzed had at least one of these four mutations. Mean variant allele frequency (VAF) of *TERT* c.-124C > T, *TERT* c.-146C > T, *ATM* c.1236-2A > T and *TP53* c.853G > A mutations was 0.17, 0.06, 0.05 and 0.06, respectively. Overall, a statistically significant correlation between high VAF (higher than the mean) and tumor progression was found for the *TERT* c.-124C > T mutation (*p* = 0.019).

Strikingly, 11 out of 16 (69%) progressive patients harbored the *TERT* c.-124C > T mutation, with all 11 patients having a high VAF.

### CTC number for patient monitoring

CTCs were observed in all patients at time of cystectomy and during follow-up. Overall, the mean CTC number (range) at time of cystectomy and one, four and 12 months after RC was 35 (11–108), 35 (18–104), 33 (15–56) and 46 (12–132) CTCs per 7.5 mL blood, respectively.

**Fig. 1:**
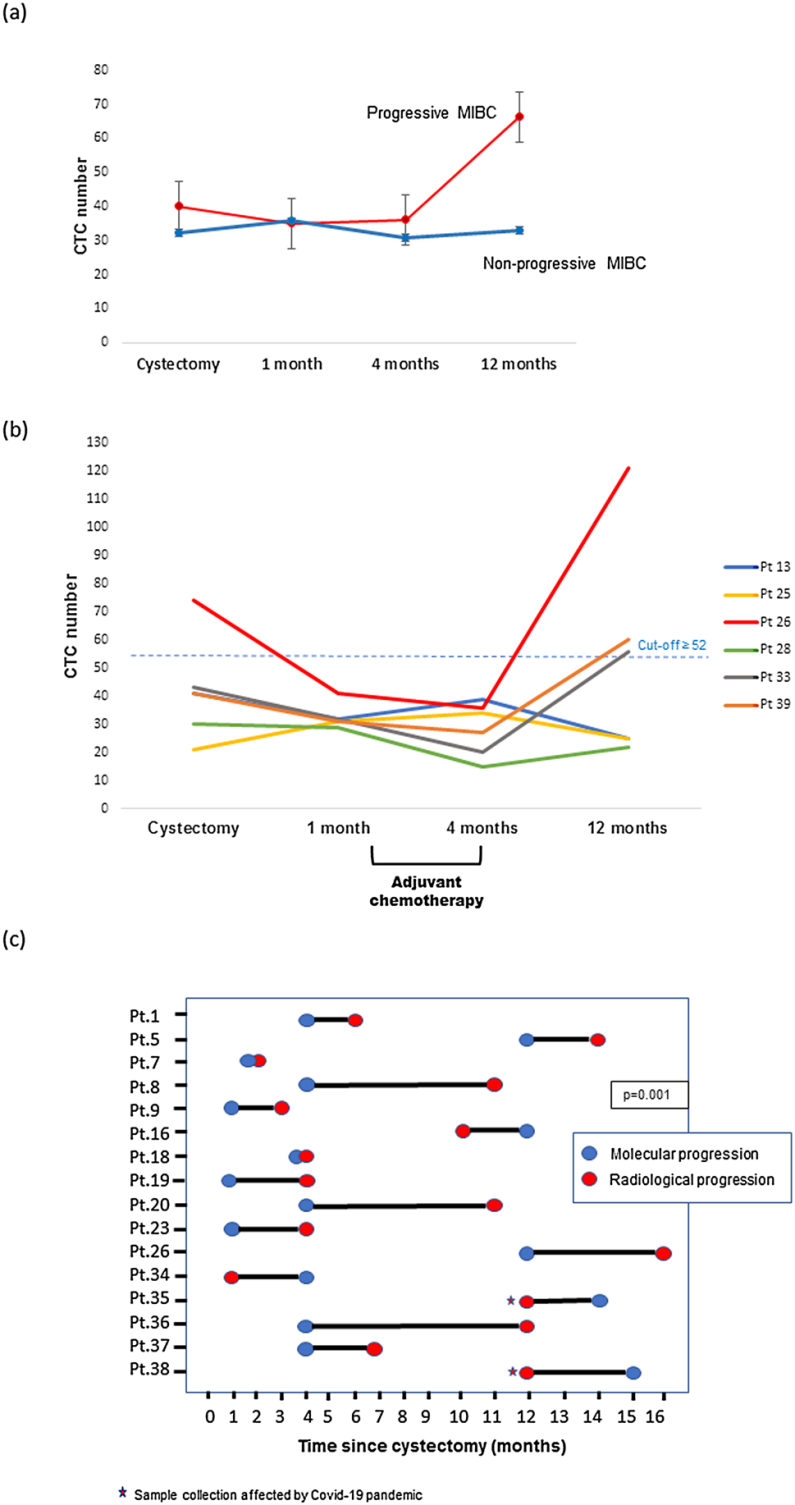
CTC enumeration in MIBC monitoring. Mean CTC number is shown at four different time points **A** for progressive and non-progressive MIBC patients and **B** for MIBC patients who underwent adjuvant chemotherapy (patients with CTC number ≥ 52 CTCs per 7.5 mL blood are considered at high risk of progression). **C** Time of progression according to CTC number (molecular progression) and imaging techniques (radiological progression). Molecular progression is considered when an increase of at least 10 CTCs per 7.5 mL blood is observed between two consecutive time points. *Pt* patient

CTC enumeration results and clinical variables during patient follow-up are summarized in Supplementary Figure S2. CTC number was significantly higher in progressive than in non-progressive patients 12 months after RC (*p* = 0.021) (Fig. 1A); however, no statistically significant differences were observed in CTC number between both groups of patients, neither at time of cystectomy (*p* = 0.855), nor one and four months after RC (*p* = 0.228; *p* = 0.191; respectively).

Six patients from our cohort received adjuvant chemotherapy between two and four months after RC (Fig. 1B). According to CTC number, three of these patients were at high risk (≥ 52 CTCs per 7.5 mL blood; see “survival analysis”) of developing metastasis 12 months after RC. In fact, one patient (Pt 26) had a very high CTC number (121 CTCs per 7.5 mL blood) and presented clinical progression at 16 months after cystectomy. The other two patients (Pt 33 and Pt 39) had a CTC number close to the cut-off (56 and 60 CTCs per 7.5 mL blood); after a median follow-up of 24 months they did not show clinical progression. The three patients with a CTC number below cut-off did not present clinical progression after a median follow-up of 28 months.

Overall, 63% (10/16) of relapses were predicted earlier with CTC enumeration than with imaging techniques, whereas two cases were detected simultaneously by both techniques, and, in four cases, relapse was predicted earlier by imaging techniques. In two of these four cases we were unable to collect blood samples 12 months after cystectomy due to the Covid-19 pandemic, the samples were collected later. These two patients progressed 12 months after RC, but the increase in CTCs was observed at 14 and 15 months after RC, although we believe that the CTC increase would have been observed at 12 months, as the increase in CTC number from the last time point evaluated was greater than 40 CTCs per 7.5 mL blood.

Remarkably, an increase in CTC number predicted tumor relapse with a median anticipation period over conventional imaging techniques of 120 days *versus* 255 days (lead time, 4 months), respectively (*p* = 0.001) (Fig. 1C).

### Correlation between tumor mutations and CTC enumeration

This study evaluated whether a relationship exists between gene mutation VAF and CTC enumeration after RC and at several follow-up time points. Overall, we found that MIBC patients harboring high VAF of the *TERT* c.-124c > T mutation (*N* = 14) presented a CTC number above the cut-off (≥ 52 CTCs per 7.5 mL blood; see “survival analysis”) 12 months after RC (*p* = 0.03). The remaining alterations (*TERT* c.-146C > T, *TP53* c. 853G > A and *ATM* c.1236-2A > T) had no significant correlation with CTC enumeration.

Notably, progressive patients harboring the *TERT* c.-124C > T mutation (69%; 11/16) presented a significant increase in CTC number 12 months after RC compared with those progressive patients without a mutation (*p* = 0.014).

### Survival analysis

Tumor tissue DNA mutations, CTC enumeration at each follow-up time point and clinical variables (pathological stage, lymph node status, chemotherapy) were evaluated by Cox regression analysis. Univariate analysis demonstrated that CTC number 12 months after RC and pathological stage are significant predictors of tumor progression. CTC number 12 months after RC and VAF of the *TERT* c.-124C > T mutation were significant predictors of cancer specific survival (CSS). Multivariate Cox regression analysis, including VAF of the *TERT* c.-124C > T mutation, CTC number at 12 months and pathological stage as covariables, identified CTC number 12 months after RC as an independent prognostic biomarker for tumor progression (HR 1.020; *p* = 0.009) and CSS (HR 1.021; *p* = 0.021).

**Fig. 2:**
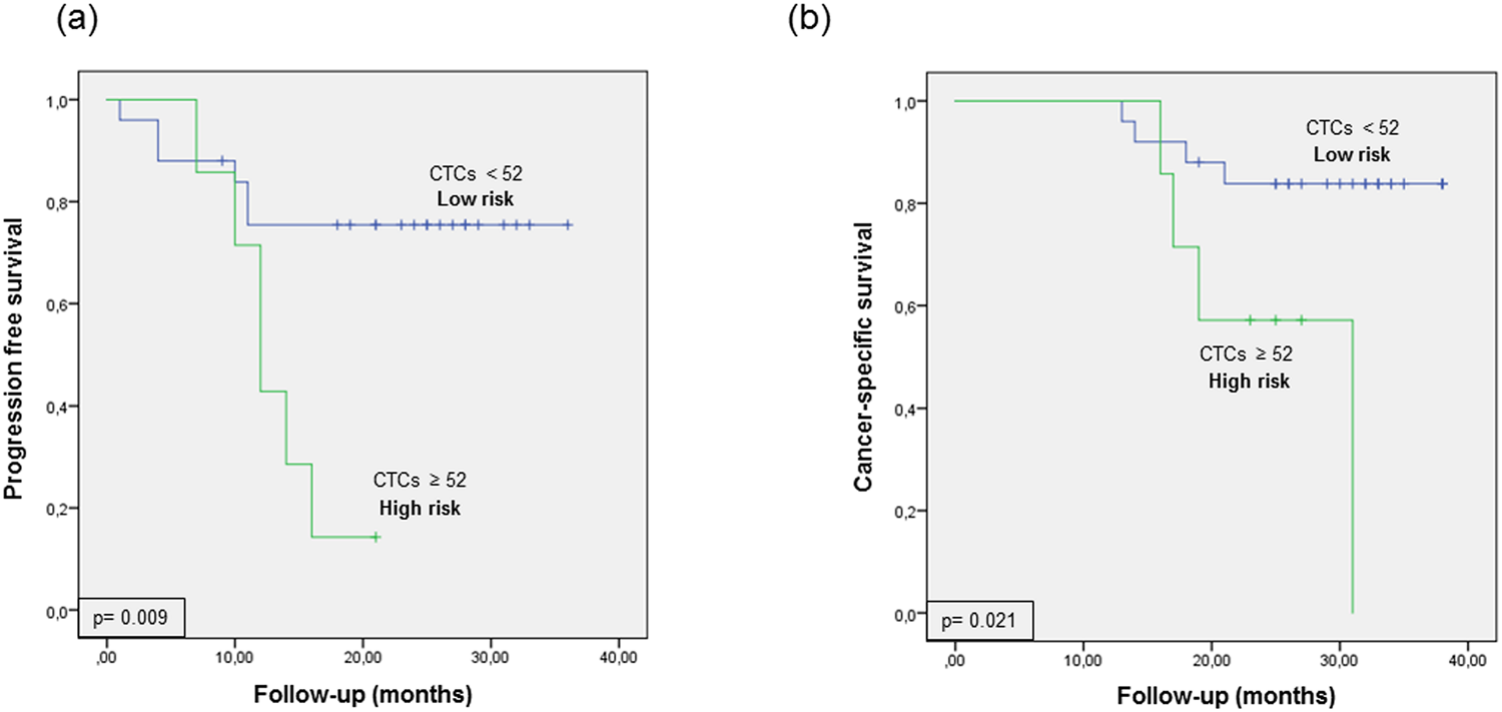
Kaplan–Meier curve for **A** tumor progression and **B** cancer specific survival (CSS) according to CTC enumeration

The mean CTC number 12 months after RC (≥ 52 CTCs per 7.5 mL blood) was used as a cut-off point to classify patients into high- and low-risk groups for tumor progression and CSS. Figure 2 shows the Kaplan–Meier curves generated using the selected cut-off point, showing that this cut-off point can discriminate between two groups of MIBC patients with a significantly different probability of tumor progression and CSS.

## Discussion

In recent years, the molecular characterization of BC has considerably advanced our understanding of BC tumor biology; however, to date, there is no prognostic biomarker established in clinical practice to identify and monitor those MIBC patients at high risk of progression after RC. To the best of our knowledge, this is the first study to evaluate DNA mutations in MIBC, enumerate blood CTCs during follow-up of patients after RC and to combine them to identify prognostic biomarkers and improve MIBC management.

In our cohort of MIBC patients, the most frequently found mutations in tumors were *TERT* c.-124C > T, *TERT* c.-146C > T, *ATM* c.1236-2A > T and *TP53* c.853G > A. *TERT* promoter mutations c.-124C > T and c.-146C > T have been frequently described in BC [9, 10] and several other tumors [11, 12]. *TERT* mutations have been detected in all stages of BC and distant metastasis, suggesting an important role of telomerase activity in the tumorigenesis of BC [9]. Mutations in the *TP53* and *ATM* genes have also been previously described in BC [13], although, as far as we know, the two specific mutations found in our cohort have never been described in BC. Of note, the *TP53* c.853G > A mutation has been found as a germline mutation in breast and lung tumors [14, 15] associated with an increased risk of cancer. Interestingly, at least one of these four mutations were present in 92% of MIBC samples, with *TERT* c.-124C > T being the most frequent (66% of patients). Furthermore, a high VAF for the *TERT* c.-124C > T mutation correlates with tumor progression. Of note, this mutation was overrepresented in the subset of progressive compared with non-progressive patients, showing a high VAF in all progressive patients. According to our results, the *TERT* c.-124C > T mutation has been previously described as a biomarker of recurrence and progression in BC [9, 10, 16]. Overall, these findings suggest that the presence of the *TERT* c.-124C > T mutation could play a crucial role in the aggressiveness of BC.

In the present study, we also found that progressive MIBC patients presented a significant increase in the mean CTC number 12 months after RC. It has been described that CTC number increases with the higher tumor burden [17–19]. Thus, CTC number could be used as a biomarker of tumor burden during follow-up of MIBC patients. Furthermore, CTCs number 12 months after RC was able to distinguish two groups of patients with a different probability of progression and CSS. A comparable result was obtained by Rink et al. [20] and Zhang et al. [21] who concluded that CTC number is a predictor of tumor progression and CSS in MIBC.

Interestingly, we showed that CTC enumeration could be a useful tool to monitor tumor relapse after systemic therapy. This result should be taken with caution, since the subset of patients from our cohort who underwent adjuvant chemotherapy is limited; however, other authors previously reported that CTC enumeration was useful for evaluating the efficacy of treatments in cancer patients [22, 23].

On the other hand, we observed a significant increase in CTC number a median of four months before radiological progression in 63% of progressive patients. A similar result was found by Christensen et al. [24] but by analyzing circulating tumor DNA instead of CTCs in a series of 99 MIBC patients. This result emphasizes the utilization of liquid biopsy for early detection of tumor progression during MIBC patient follow-up.

Finally, the correlation between mutation analyses and CTC enumeration indicated that progressive patients with a high VAF of the *TERT* c.-124C > T mutation presented a CTC number exceeding the cut-off 12 months after RC, reinforcing the fact that the *TERT* c.-124C > T mutation could be considered a biomarker of aggressivity in BC.

The relevance of the present work lies in the fact that it is the first to combine tumor mutational analysis with CTC enumeration at different time points during follow-up of MIBC patients after RC. Our results suggest that those patients harboring tumors with high VAF of the *TERT* c.-124C > T mutation are at higher risk of progression and, therefore, could be optimal candidates for closer follow-up via CTC enumeration. It is important to note that the methodology used to analyze mutations and CTCs is easily available in diagnostic laboratories, reasonably simple and has a higher sensitivity than other technologies such as the FDA approved CellSearch system [22, 25–27] and, thus, it can be easily implemented in clinical practice. However, we must acknowledge some study limitations, such as the high economic cost of isolating CTCs, the series size (although our series has been represented by a total of 150 samples analyzed) and the low number of patients who received chemotherapy. A final validation of our results in a larger and independent series is necessary to define the real role of the *TERT* c.-124C > T mutation and CTC enumeration as prognostic biomarkers in MIBC patients after RC.

## Conclusion

The presence of the *TERT* c.-124C > T mutation in muscle-invasive bladder tumor could be considered a biomarker of tumor aggressivity. CTC enumeration in the bloodstream of MIBC patients after RC is a useful tool to identify patients with a high risk of progression and CSS during follow-up and to detect tumor progression earlier than imaging techniques. MIBC patients harboring a high VAF of the *TERT* c.-124C > T mutation and high CTC number in the follow-up had a greater risk of progression. The implementation of this combined non-invasive tool in the clinical setting could have an impact on disease management since patients could benefit from early treatment.

## Supplementary Information

Below is the link to the electronic supplementary material.

Supplementary file1 (DOCX 6901 KB)

Supplementary file2 (XLSX 40 KB)

## Abbreviations

BC: Bladder cancer
CSS: Cancer specific survival
CTC: Circulating tumor cell
ctDNA: Circulating tumor DNA
EMT: Epithelial-mesenchymal transition
LN: Lymph nodes
MIBC: Muscle-invasive bladder cancer
NAC: Neoadjuvant chemotherapy
NGS: Next generation sequencing
PBMC: Peripheral blood mononuclear cell
RC: Radical cystectomy
TERT: Telomerase reverse transcriptase
TURBT: Transurethral resection of bladder tumor
VAF: Variant allele frequency
