# Supplementary material for: Prognostic implication of TERT promoter mutation and circulating tumor cells in muscle-invasive bladder cancer
Source: World J Urol. 2022 Jun 17;40(8):2033–9. doi: 10.1007/s00345-022-04061-9 (PMC9203260; doi:10.1007/s00345-022-04061-9)

**WORLD JOURNAL OF UROLOGY**

**Prognostic implication of *TERT* promoter mutation and circulating tumor cells in muscle-invasive bladder cancer**

Raquel Carrasco ¹, Mercedes Ingelmo-Torres ¹, Ascensión Gómez ¹, Fiorella L. Roldán ¹, Natalia Segura ¹, María José Ribal ¹, Antonio Alcaraz ¹, Laura Izquierdo ¹^,¥^ and Lourdes Mengual ^1,2^

^1^ Laboratory and Department of Urology, Hospital Clínic, Institut d’Investigacions Biomèdiques August Pi i Sunyer (IDIBAPS), University of Barcelona, Barcelona, Spain.

^2^ Department of Biomedical Sciences, Faculty of Medicine and Health Sciences, University of Barcelona, Barcelona, Spain.

*^¥^Corresponding author*

**Contact information corresponding author**

Laura Izquierdo, M.D., Ph.D.

Laboratory and Department of Urology. Hospital Clínic de Barcelona

Centre de Recerca Biomèdica CELLEX, office B22.

C/Casanova, 143

08036 Barcelona, Spain

Tel: (+34) 93 227 54 00 Ext. 4820

e-mail: [lizquier@clinic.cat](mailto:lizquier@clinic.cat)

**SUPPLEMENTARY MATERIAL**

**MATERIAL AND METHODS**

***Patients and samples***

All patients had localized or locally advanced MIBC at time of diagnosis. Exclusion criteria were presence of another active neoplasm.

Tumor dissemination was controlled postoperatively by computed tomography scan at 3-month intervals for the first year, 6-month intervals for the next two years and annually thereafter. Tumors were considered progressing when relapse or distant metastasis developed during follow-up.

***Tissue specimens and DNA isolation***

Formalin-fixed paraffin-embedded (FFPE) tissue sections of 20 µm were obtained from the IDIBAPS biobank. Genomic DNA (gDNA) was isolated from FFPE sections using the RecoverAll Total Nucleic Acid Isolation kit (Ambion, Inc. Austin, TX, USA), according to manufacturer’s instructions. gDNA was quantified by spectrophotometric analysis at 260 nm (NanoDrop Technologies, Wilmington, DE, USA).

***Next generation sequencing (NGS) of tumoral tissue***

Ion Torrent Oncomine Comprehensive Assay v3 (Thermo Fisher Scientific, Massachusetts, USA) is a panel based on Ion Ampliseq and covers 161 of the most relevant cancer driver genes (Supplementary Table S2). Ten ng of DNA was used for library preparation according to manufacturer’s instructions. Sequencing was performed on an Ion S5 System using the Ion 540 Chip (Thermo Fisher Scientific).

***Blood sample processing and circulating tumor cell (CTC) isolation***

The peripheral blood mononuclear cell (PBMC) fraction was isolated with Leucosep tubes containing 15 mL of Ficoll-Plaque Plus, 7.5 mL of blood sample and 7.5 mL of PBS 1X. Leucosep tubes were centrifuged 20 min at room temperature and the PBMC fraction recovered. PBMCs were processed using the CTC Enrichment Kit (Fluxion, Biosciences Inc, San Francisco, California, United States), according to manufacturer’s instructions. The kit contains immunomagnetic beads that are pre-conjugated with specific antibodies to recover epithelial and mesenchymal cells (EpCAM, EGFR, Mesenchymal markers).

***CTC isolation and enumeration***

CTC Enumeration Kit (Fluxion, Biosciences) includes anti-CK-fluorescein isothiocyanate (FITC; specific for intracellular cytokeratin of epithelial cells), anti-CD45- Indocarbocyanine (Cy3; specific for leukocytes) and Hoechst 33342 (which stains the cell nucleus). CTCs are defined as morphologically intact, CK positive, CD45 negative and nucleated cells (Supplementary Figure S1). Number of CTCs are referred to 7.5 mL blood.

***Statistical analysis***

DNA mutations were dichotomized using the mean value of variant allele frequency (VAF). Correlations between DNA mutations and 1) CTC enumeration at different follow-up time points, 2) tumor progression, 3) cancer specific survival (CSS) and 4) pathological stage were analyzed using the Spearman’s Rank-Order Correlation (Spearman’s Rho). Kaplan–Meier curves were generated and compared using log-rank tests to examine the influence of VAF on tumor progression and CSS.

Assessment of differences in CTC number between progressive and non-progressive patients during follow-up was analyzed using the Mann-Whitney U-test for independent samples. The Wilcoxon test was used for analyzing CTC increase before radiological progression. Cox regression analysis was performed to examine influence of CTC number on tumor progression and CSS at different follow-up time points. CTC number was dichotomized using the mean value of CTC number at each follow-up time point. Thereafter, Kaplan–Meier curves were generated and compared using the log-rank test to examine the influence of CTC number on tumor progression and CSS.

Statistical significance was established at a p-value of 0.05. All analyses were carried out with the SPSS software package (IMB SPSS Statistics 23).

**SUPPLEMENTARY TABLES AND FIGURES**

**Supplementary Table S1. Clinicopathological features of muscle-invasive bladder cancer (MIBC) patients.**

|  |  | **TOTAL MIBC**  **(N=39)** | **Progressive MIBC (N=16)** | **Nonprogressive MIBC (N=23)** |
| --- | --- | --- | --- | --- |
|  |  | N (%) | N (%) | N (%) |
| Gender | |  |  |  |
| Male | | 31 (79.5) | 12 (75) | 19 (82.6) |
| Female | | 8 (20.5) | 4 (25) | 4 (17.4) |
| Median Age | | 70 | 70 | 71 |
| Pathological Stage | |  |  |  |
| pT0, pTa, pT1, pTIS | | 10 (25.6) | 2 (12.5) | 8 (34.7) |
| pT2 | | 8 (20.5) | 3 (18.8) | 5 (21.8) |
| pT3 | | 15 (38.5) | 6 (37.5) | 9 (39.1) |
| pT4 | | 6 (15.4) | 5 (31.2) | 1 (4.4) |
| Lymph Nodes (LN) | |  |  |  |
| LN+ | | 4 (10.3) | 2 (12.6) | 2 (8.8) |
| pT2 | | *1 (2.6)* | *-* | *1 (4.4)* |
| pT3 | | *2 (5.1)* | *1 (6.3)* | *1 (4.4)* |
| pT4 | | *1 (2.6)* | *1 (6.3)* | *-* |
| LN- | | 35 (89.7) | 14 (87.4) | 21 (91.2) |
| Neoadjuvant Chemotherapy | | 7 (17.9) | 4 (25.1) | 3 (13.1) |
| pT0, pTa, pT1, pTIS | | *2 (5.1)* | *1 (6.3)* | *1 (4.4)* |
| pT2 | | *-* | *-* | *-* |
| pT3 | | *3 (7.7)* | *1 (6.3)* | *2 (8.7)* |
| pT4 | | *2 (5.1)* | *2 (12.5)* | *-* |
| Adjuvant Chemotherapy | | 6 (15.4) | 1 (6.3) | 5 (21.8) |
| pT0, pTa, pT1, pTIS | | *-* | *-* | *-* |
| pT2 | | *1 (2.6)* | *-* | *1 (4.4)* |
| pT3 | | *3 (7.7)* | *-* | *3 (13)* |
| pT4 | | *2 (5.1)* | *1 (6.3)* | *1 (4.4)* |

**Supplementary Table S2. List of gene targets in Oncomine Comprehensive Assay v3 (Thermo Fisher Scientific).**

| Hotspot genes | | | | Full-length genes | | Copy number genes | | Genes fusions (inter- and intragenic) | |
| --- | --- | --- | --- | --- | --- | --- | --- | --- | --- |
| *AKT1*  *ALK*  *AR*  *ARAF BRAF*  *BTK*  *CBL*  *CDK4 CHEK2 CSF1R CTNNB1 DDR2 EGFR ERBB2 ERB83 ERBB4 ESR1 EZH2 FGFR1 FGFR2 FGFR3 FLT3*  *FOXL2 GATA2 GNA11 GNAQ* | *GNAS HNF1A HRAS*  *IDH1*  *IDH2*  *JAK1*  *JAK2*  *JAK3*  *KDR*  *KIT*  *KNSTRN KRAS MAGOH MAP2K1 MAP2K2 MAPK1 MAX MED12*  *MET*  *MTOR MYD88 NFE2L2 NRAS PDGFRA PIK3CA*  *PPP2R1A* | *PTPN11 RAC1*  *RAF1*  *RET*  *RHEB*  *RHOA SF3B1*  *SMO*  *SPOP*  *SRC*  *STAT3 U2AF1*  *XPO1*  *AKT2 AKT3*  *AXL CCND1 CDK6 ERCC2 FGFR4 H3F3A*  *HIST1H3B MAP2K4 MDM4 MYC MYCN* | *NTRK1 NTRK2 PDGFRB PIK3CB ROS1 SMAD4 TERT TOP1* | *ATM*  *BAP1*  *BRCA1 BRCA2 CDKN2A FBXW7 MSH2*  *NF1*  *NF2*  *NOTCH1 PIK3R1 PTCH1*  *PTEN*  *RB1*  *SMARCB1 STK11*  *TP53*  *TSC1*  *TSC2 ARID1A*  *ATR*  *ATRX*  *CDK12 CDKN1B CDKN2B*  *CHEK1* | *CREBBP FANCA FANCD2 FANCI MLH1 MRE11A*  *MSH6 NBN NOTCH2 NOTCH3 PALB2 PMS2 POLE RAD50 RAD51 RAD51B RAD51C RAD51D RNF43 SETD2 SLX4 SMARCA4* | *AKT1*  *AR*  *CCND1 CCNE1 CDK4*  *CDK6 EGFR ERBB2 FGFR1 FGFR2 FGFR3 FGFR4 FLT3*  *IGF1R*  *KIT*  *KRAS MDM2 MDM4*  *MET*  *MYC*  *MYCL MYCN PDGFRA PIK3CA*  *PPARG*  *TERT* | *AKT2*  *AKT3*  *ALK*  *AXL*  *BRAF*  *CCND2 CCND3*  *CDK2*  *CDKN2A CDKN2B ESR1*  *FGF19*  *FGF3*  *NTRK1 NTRK2 NTRK3 PDGFRB PIK3CB RICTOR TSC1*  *TSC2* | *ALK*  *AXL BRAF EGFR ERBB2 ERG ETV1 ETV4 ETV5 FGFR1 FGFR2 FGFR3 NTRK1 NTRK3*  *PDGFRA PPARG RAF1*  *RET*  *ROS1*  *AKT2*  *AR*  *BRCA1*  *BRCA2 CDKN2A ERB84 ESR1* | *FGR FLT3 JAK2 KRAS MDM4 MET MYB MYBL1*  *NF1 NOTCH1 NOTCH4 NRG1 NTRK2 NUTM1 PDGFRB PIK3CA PRKACA PRKACB PTEN RAD51B RB1 RELA RSPO2 RSPO3 TERT* |

**Supplementary Table S3. Mutations presented in tumor samples detected by NGS.**

Abbreviation: Pt – Patient; SNV – Single-Nucleotide Variant; CNV – Copy Number Variant; INDEL – Insertion and Deletion

(See excel file).

**Supplementary Figure S1. Immunofluorescent staining of CTCs.** White arrows show a CTC: Anti-CK-FITC positive, Anti-CD45-Cy3 negative and Hoechst 33342 positive. (Original picture)


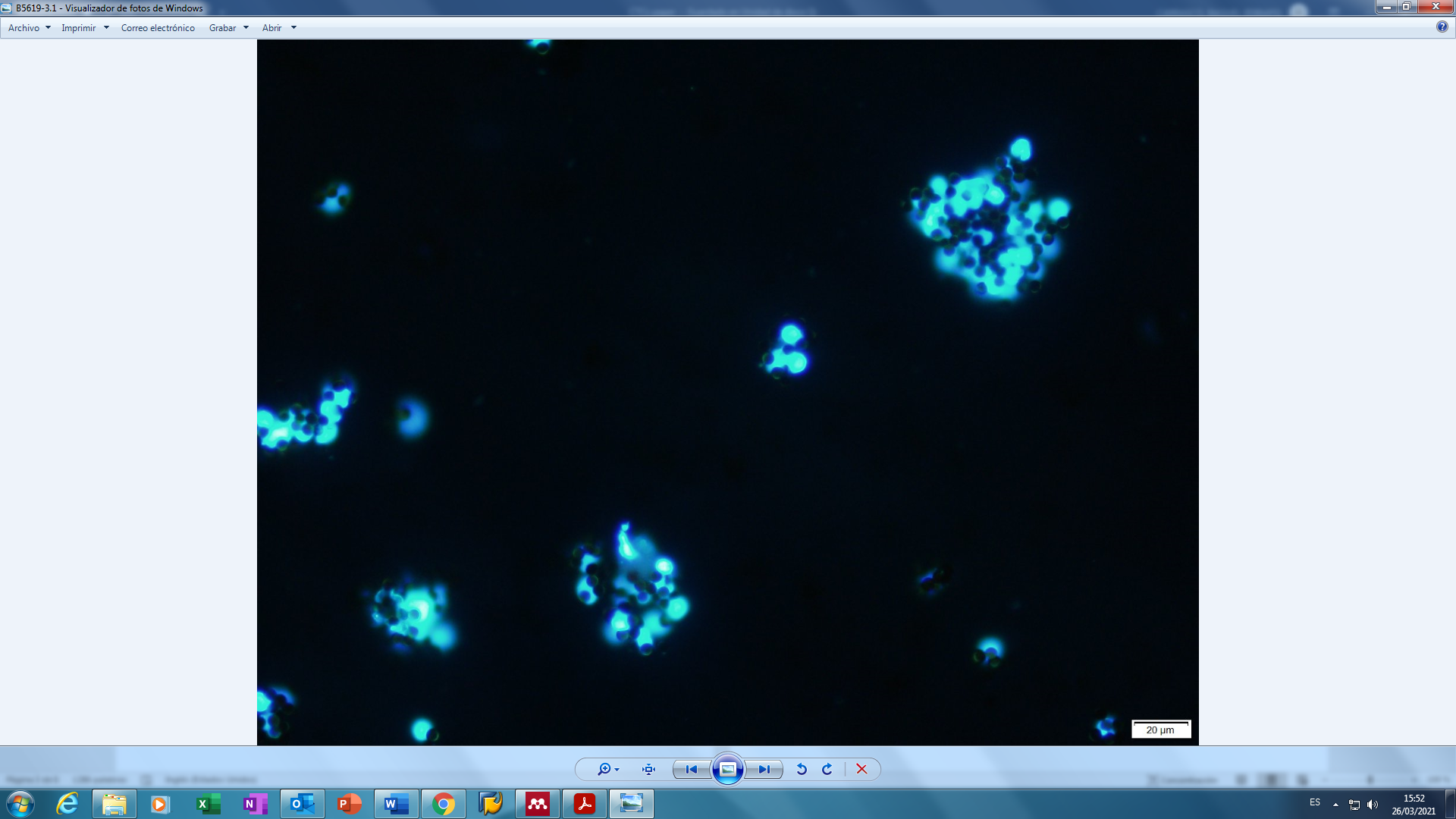

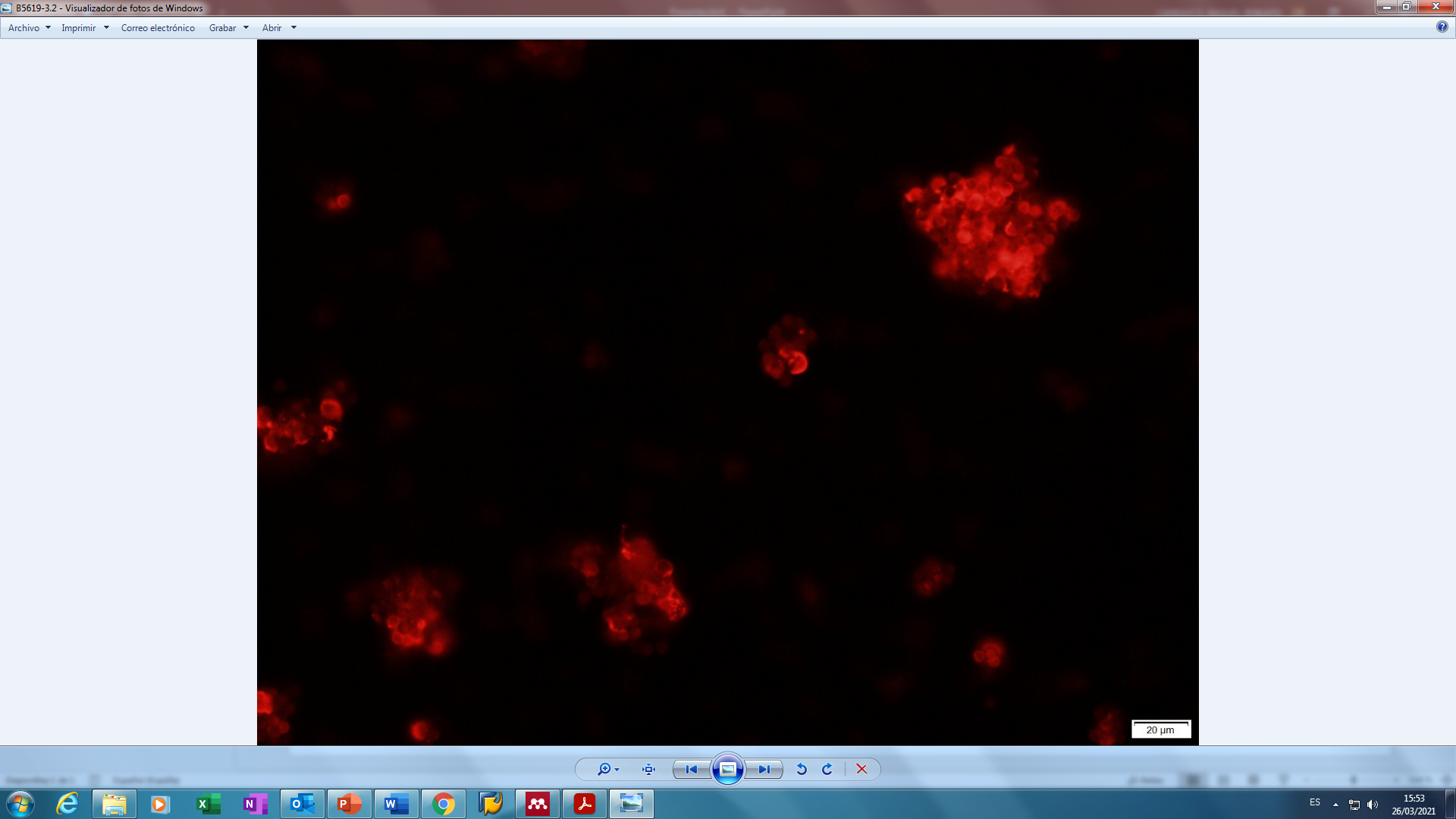

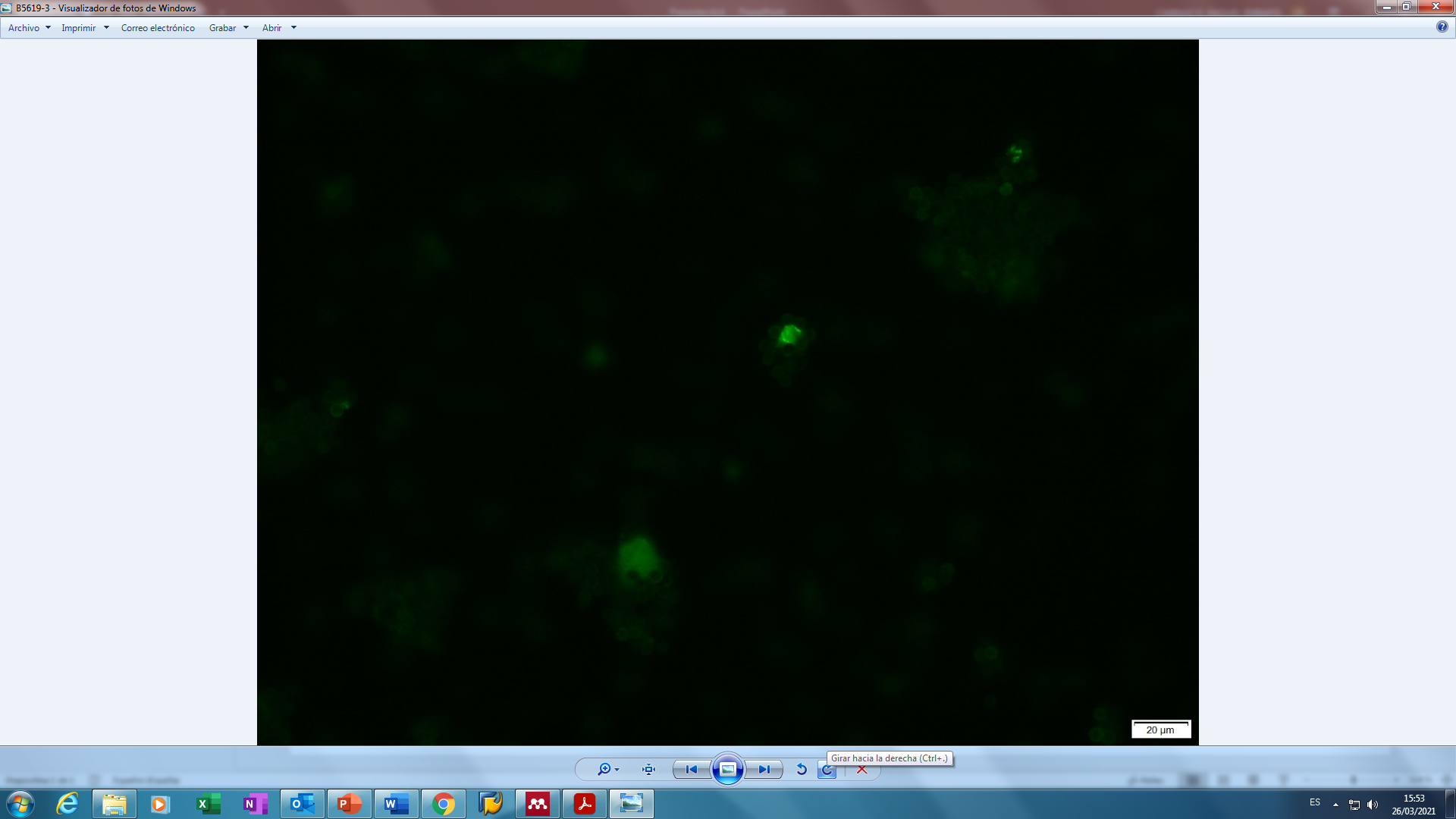


**Anti-CK-FITC +**

**Anti-CD45-Cy3 -**

**Hoechst 33342**

**(Nucleated)**

**Supplementary Figure S2. Liquid biopsy analysis summary.** Graphical overview of circulating tumor cell (CTC) enumeration analyses and clinical variables for the 39 muscle-invasive bladder cancer (MIBC) patients included in the study. Each line represents a patient with circles indicating the CTC number range to the timeline presented at the top. Patients with CTC number ≥ 52 CTCs per 7.5 mL blood (red circles) are considered at high risk of progression. Grouping of patients into “Non-progressive” and “Progressive” was based on radiographic imaging results at controls during the follow-up.

Abbreviation: Pt.- Patient


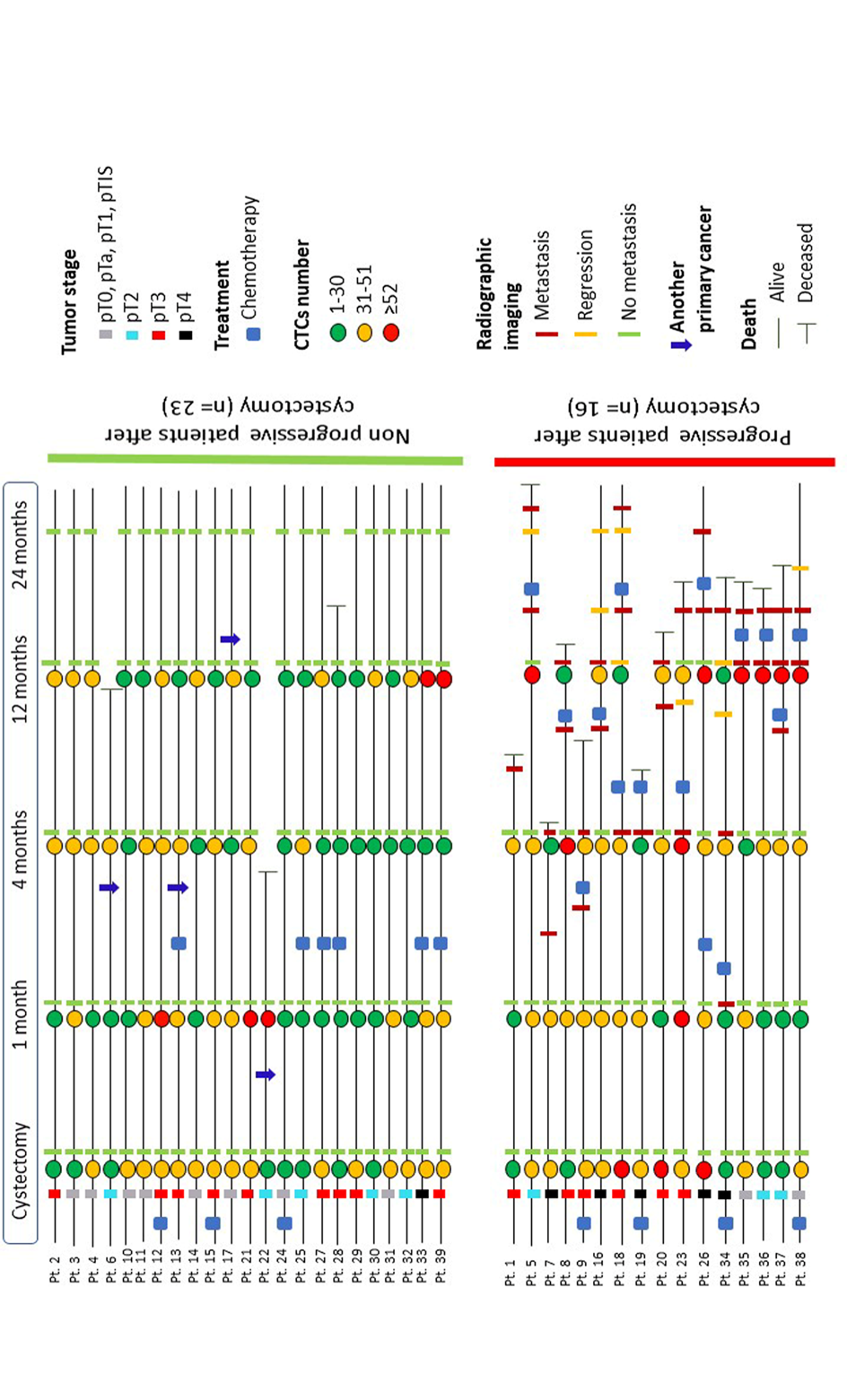

Supplement: Supplementary file 1 — Supplementary file1 (DOCX 6901 KB) [file 345_2022_4061_MOESM1_ESM.docx]
